# Characteristics of the gut microbiome of asymptomatic hyperuricemia

**DOI:** 10.3389/fendo.2025.1557225

**Published:** 2025-10-15

**Authors:** Fengjiao Cao, Wenming Yi, Mengwei Wu, Ao Gao, Tianlun Kang, Xiujuan Hou

**Affiliations:** ^1^ Department of Preventive Treatment of Disease, Dongfang Hospital, Beijing University of Chinese Medicine, Beijing, China; ^2^ Department of Rheumatology, Dongfang Hospital, Beijing University of Chinese Medicine, Beijing, China

**Keywords:** asymptomatic hyperuricemia, gut microbiota, 16S rRNA sequencing, correlation study, clinical parameters

## Abstract

**Purpose:**

Asymptomatic hyperuricemia(AH) is characterized by elevated blood uric acid levels without symptoms,posing risks like gout, kidney stones, and cardiovascular diseases. This study aims to investigate the role of the gut microbiota in uric acid metabolism in AH.

**Methods:**

Clinical data from 30 AH patients and 30 healthy controls were collected. Fecal microbiota genomic DNA was extracted, PCR amplified, library constructed, and sequenced. Bioinformatics and statistical analyses were conducted to study the gut microbiota of the two groups.

**Results:**

The AH group exhibited significantly elevated levels of body mass index (BMI), Triglycerides (TG), Total Cholesterol (TC), as along with a history of smoking, hypertension, and fatty liver disease compared to the healthy group (P < 0.05). The overall richness and ecological diversity of gut microbiota in the AH group decreased, with differences in the distribution at the phylum and genus levels compared to the healthy group. Uric acid demonstrated significant correlations with various gut microbiota (e.g., Granulicatella), suggesting their potential as biomarkers for AH. Despite limitations such as a small sample size and lack of long-term follow-up, our findings provide new insights for the early diagnosis and personalized treatment of AH. Looking ahead, these discoveries may advance the clinical management of AH and the exploration of associated biomarkers.

## 1 Introduction

Asymptomatic hyperuricemia(AH) is characterized by elevated levels of uric acid in the blood without clinical symptoms. Despite its increasing prevalence in adults, the potential health risks associated with this condition are often overlooked (1). AH is closely linked to the development of gout, kidney stones, and cardiovascular diseases (2), imposing significant economic burdens on healthcare systems. Current diagnostic and treatment approaches primarily focus on symptomatic gout patients, while prevention and management of AH lack sufficient research and attention, highlighting the urgent need for further exploration and investigation in this field.

Recent research has indicated a potential significant role of the gut microbiota in metabolic disorders, including the regulation of uric acid metabolism (3, 4). The composition of the gut microbiota is closely associated with an individual’s metabolic status, with certain microbes potentially impacting the synthesis and excretion of uric acid (5). This discovery has sparked scientific interest in exploring the connection between AH and the gut microbiota, suggesting a potential significant role of the gut microbiota in the development of hyperuricemia (6). Therefore, research focusing on the gut microbiota may unveil new pathophysiological insights into AH and offer novel avenues for intervention.

This study aims to explore the potential connection between gut microbiota characteristics and uric acid metabolism in patients with AH. The research methods include clinical data collection, DNA extraction and sequencing, microbiome data analysis, to elucidate how microbiota composition impacts the development and advancement of AH. This not only provides foundational data for mechanistic studies of AH but also offers potential biomarker support for future personalized treatment strategies.

Upon reviewing the background and existing research, it is evident that this study will provide a new perspective on the prevention and treatment of AH, particularly in the individualized intervention of the microbiome. This will offer a more scientific basis for clinical practice, ultimately reducing the incidence of AH and its related complications, thereby improving patients’ quality of life.

## 2 Materials and methods

### 2.1 Study population

In the period from January to June 2023, we recruited 30 patients with AH at the Health Examination Center of Dongfang Hospital, Beijing University of Chinese Medicine. The diagnostic criteria for AH were defined as serum uric acid levels of > 7 mg/dL for males and > 6 mg/dL for females in two fasting tests on different dates under normal purine diet (7). Exclusion criteria for the AH patients included: a history of acute gouty arthritis, chronic tophaceous gout, chronic gouty arthritis, or uric acid nephropathy; the presence of severe cardiovascular, cerebrovascular, hepatic, renal, or hematopoietic system diseases; and secondary hyperuricemia due to other causes such as malignancy or renal disease. Additionally, we excluded individuals who had taken medications known to influence uric acid metabolism (e.g., aspirin, hydrochlorothiazide, probenecid) or those who had used antibiotics, probiotics, prebiotics, or synbiotics within the 3 months prior to enrollment. Pregnant or lactating women were also excluded. A parallel group of 30 age- and gender-matched healthy volunteers was recruited. These volunteers had no history of significant diseases or infections in the past 3 months and had likewise not used any antibiotics or probiotic supplements during that period.

Sample size estimation: The sample size was determined *a priori* using power analysis in G*Power 3.1, where based on pilot data from 5 asymptomatic hyperuricemia (AH) patients and 5 healthy controls, we estimated a Cohen’s d effect size of 0.80 for α-diversity (Shannon index) differences; with a significance level (α) of 0.05 (two-tailed) and 80% statistical power, the minimum required sample was calculated as 22 participants per group, which was increased to 30/group to accommodate 20% potential attrition from DNA extraction/sequencing failures.

This study was conducted in accordance with the Helsinki Declaration and Good Clinical Practice guidelines. Approved by the Ethics Committee of Dongfang Hospital, Beijing University of Chinese Medicine (Approval No: JDF-IRB-2023051802), all participants provided written informed consent.

### 2.2 Clinical information

Patient consultation and physical examination data were collected, including patient ID, gender, age, height, weight, liver ultrasound results, medical history, smoking and alcohol consumption history. Body mass index (BMI) was calculated using the formula BMI = weight (kg)/height (m^2) based on the patient’s height and weight information. Blood pressure was measured using a clinical electronic sphygmomanometer, with two seated blood pressure measurements taken 10 minutes apart and the average recorded. Hypertension was defined as systolic blood pressure ≥ 140 mmHg, diastolic blood pressure ≥ 90 mmHg, a history of hypertension, and/or current use of antihypertensive medication. Diabetes was defined as fasting blood glucose ≥ 7.1 mmol/l, a history of diabetes, and/or current use of antidiabetic medication. Smoking history referred to current or past smoking habits, while alcohol consumption history referred to the consumption of alcoholic beverages in the past year. Fatty liver was diagnosed based on abdominal ultrasound findings or a history of fatty liver disease. Additionally, 5ml of peripheral venous blood was collected from each patient after a 12-hour fast for laboratory testing, including routine blood parameters, blood biochemistry, renal and liver function parameters, and blood lipid analysis.

### 2.3 Specimen collection

Before sampling, instruct the participants to empty their bladders to prevent urine contamination of feces. Collect approximately 2g of freshly passed feces using a sterile spoon and place it in a 2 mL cryotube. Store the sample at -80 °C within 2 hours for sequencing.

### 2.4 DNA extraction and PCR amplification

In this study, genomic DNA of fecal gut microbiota was extracted using the CTAB method. Subsequently, DNA purity and concentration were assessed by 1% agarose gel electrophoresis. An appropriate amount of fecal sample was diluted in sterile water to 1 ng/µl in a centrifuge tube. The highly variable V3V4 region of the bacterial 16S rRNA gene was selected for sequencing. Specific primers 341F (5’-CCTAYGGGRBGCASCAG-3’) and 806R (5’-GGACTACNNGGGTATCTAAT-3’) were used for PCR amplification of the V3 + V4 variable region. The amplification protocol involved using the diluted genomic DNA as a template, specific primers designed with specific barcodes, Phusion^®^ High-Fidelity DNA polymerase enzyme, and Phusion^®^ High-Fidelity PCR Master Mix with GC Buffer. Amplification of the V3 + V4 variable region was carried out using a Bio-rad T100 gradient PCR machine.

### 2.5 Purification and multiplexing of PCR products

After equalizing the concentrations of PCR products, they were thoroughly mixed and purified using a 2% agarose gel electrophoresis in 1xTAE buffer (Biowest, Spain). The target bands were recovered using the Universal DNA Purification Recovery Kit (TianGen, China).

### 2.6 Construction of libraries and sequencing on computers

Utilizing the NEB Next^®^ Ultra DNA Library Prep Kit (Illumina, USA), libraries were constructed, followed by library quality assessment and qPCR quantification using the Agilent 5400 Bioanalyzer (Agilent Technologies, USA). Subsequently, qualified libraries were subjected to sequencing on the Illumina Novaseq 6000 PE250 platform (Illumina, USA).

### 2.7 Bioinformatics analysis

The analysis was conducted by following the “Atacama soil microbiome tutorial” of Qiime2docs along with customized program scripts (https://docs.qiime2.org/2019.1/).

Utilizing the QIIME2 dada2 plugin, all raw sequences underwent quality control, trimming, denoising, merging, and removal of chimeras to obtain the final feature sequences (amplicon sequence variants, ASVs). The ASV representative sequences were aligned to the pre-trained 13_8 version of the GREENGENES database at 99% similarity using the QIIME2 feature-classifier plugin (with the database trimmed to the V3V4 region based on the 341F/806R primers), resulting in a table of taxonomic classifications. Subsequently, the QIIME2 feature-table plugin was employed to eliminate all contaminant mitochondrial and chloroplast sequences. Various methods such as ANCOM, ANOVA, Kruskal-Wallis, LEfSe, and DESeq2 were employed to identify differential bacterial abundance between groups and samples. Subsequently, the QIIME2 core-diversity plugin was utilized to compute diversity matrices, including alpha diversity indices at the feature sequence level such as observed features, Chao1, Simpson, Shannon, and Faith’s phylogenetic diversity, to assess the diversity within samples. Beta diversity indices, such as Bray Curtis, unweighted UniFrac, and weighted UniFrac, are utilized to assess differences in microbial community structures among samples. We employed Principal Coordinates Analysis (PCoA) and Partial Least-Squares Discrimination Analysis (PLS-DA) plots for visualization. To further understand the specific species contributing to inter-group microbial differences, bacteria with differential abundances between groups were identified using the Kruskal-Wallis test and Linear discriminant analysis Effect Size (LEfSe) based on species abundance tables. Spearman correlation coefficients were calculated between clinical phenotypes and microbial species, and a correlation heatmap was constructed to assess significant associations. Additionally, the functional composition of microbial communities was predicted using PICRUSt software. Unless stated otherwise, default parameters were utilized for the aforementioned analyses. (Sequencing service and data analysis service were provided by Wekemo Tech Group Co., Ltd. Shenzhen China.).

## 3 Result

### 3.1 The fundamental characteristics of the research subject.

The AH group and the healthy control group showed no significant differences in age and gender (P > 0.05). The AH group exhibited significantly higher levels of BMI, Serum Uric Acid (SUA), TG, and TC compared to the healthy control group (P < 0.05), while High-Density Lipoprotein Cholesterol (HDLC) levels were significantly lower in the AH group (P < 0.05). Differences in hypertension, fatty liver, and alcohol history were statistically significant (P < 0.05) (**Table 1**).

**Table 1:** Comparison of baseline characteristics of the participants [(x ± s/n (%)].

Clinical data	Healthy group(*n*=30)	AH group(*n*=30)	*P-value*
Gender			0.79
Male = 1	17(56.7%)	18(60%)	
Female = 0	13(43.3%)	12(40%)	
Age(years)	38.03 ± 11.35	34.43 ± 9.65	0.19
BMI(kg/m^2^)	23.36 ± 3.00	27.56 ± 4.71	0.00
SUA(mmol/L)	301 ± 67.00	462 ± 84.37	0.00
TC(mmol/L)	4.45 ± 0.75	4.96 ± 1.11	0.04
TG(mmol/L)	1.21 ± 0.61	1.97 ± 1.49	0.01
LDL-C(mmol/L)	2.80 ± 0.73	3.20 ± 0.96	0.07
HDL-C(mmol/L)	1.45± 0.34	1.21 ± 0.26	0.04
Fatty liver			
Yes = 1	5(16.7%)	19(63.3%)	0.00
No = 0	25(83.3%)	11(36.7%)	
Smoking history			
Yes = 1	6(20%)	13(43.3%)	0.52
No = 0	24(80%)	17(56.7%)	
Alcohol consumption history			
Yes = 1	6(20%)	19(63.3%)	0.00
No = 0	24(80%)	11(36.7%)	
Hypertension			
Yes = 1	2(6.7%)	11(36.7%)	0.01
No = 0	28(93.3%)	19(63.3%)	
Diabetes			
Yes = 1	4(13.3%)	10(33.3%)	0.07
No = 0	26(86.7%)	20(66.7%)	

### 3.2 The characteristics of the distribution at the phylum and genus levels of gut microbiota in two groups.

**Figure 1:**
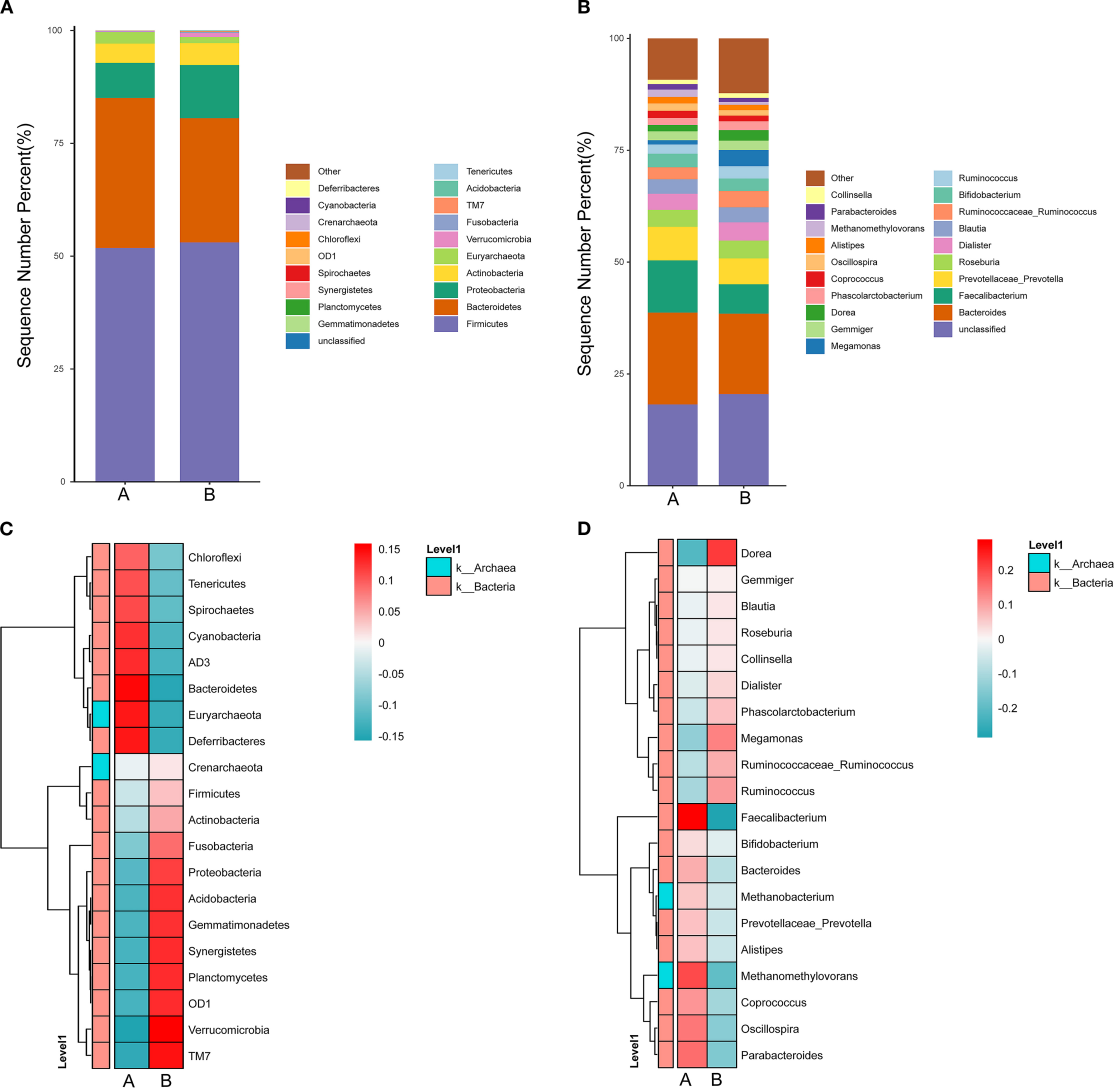
Characteristics of the distribution of gut microbiota at the phylum and genus levels in two groups. **(A)** displays the relative abundance of species at the phylum level, while **(B)** illustrates the relative abundance of species at the genus level. The x-axis represents the group names, and the y-axis (Sequence Number Percent%) indicates the proportion of sequences annotated at that level compared to the total annotated data, with the color sequence in the bar charts corresponding to the legend on the right. **(C)** represents a heatmap of species communities at the phylum level, and **(D)** shows a heatmap of species communities at the genus level. The x-axis denotes the group names, and the y-axis displays the phylum/genus-level taxonomic annotations. The clustering tree on the left clusters species based on similarity in abundance distribution. The middle heatmap represents the log10(absolute abundance) heatmap. Group A refers to the healthy group, while Group B refers to the AH group.

By analyzing the feature table of Amplicon Sequence Variants (ASVs), the relative abundances of samples at different taxonomic levels including phylum, class, order, family, genus, and species were determined. The results were presented using stacked bar graphs. At the phylum level, the top 20 species were selected to compose the bar graph, as shown in **Figure 1A**. Both groups exhibited Firmicutes, Bacteroidota, Proteobacteria, Actinobacteria, and Euryarchaeota as dominant gut microbiota. Among the phyla with relatively higher proportions, the abundance of Euryarchaeota in the AH group was significantly lower compared to the other group (2.54% vs. 1.345%, P=0.019).

At the genus level, a bar graph was constructed using the top 20 ranked species, as shown in **Figure 1B**. The gut microbiota of the two groups exhibited differences at the genus level, with relatively higher abundances in the following genera for both groups: Bacteroides (20.40% vs 20.89%), Faecalibacterium (10.74% vs 12.98%), and Prevotella (8.01% vs 10.58%). In the AH group, Oscillospira (1.63% vs 1.18%, p=0.035) and Methanomethylovorans (1.63% vs 0.65%, p=0.003) showed significantly lower abundance levels.

As shown in **Figures 1C, D**, the community heatmap illustrates the species abundance of the top 20 gut-dominant microbiota at the phylum and genus levels for two groups, revealing differences in gut microbiota composition between the groups.

### 3.3 Comparison of alpha diversity of gut microbiota

**Figure 2:**
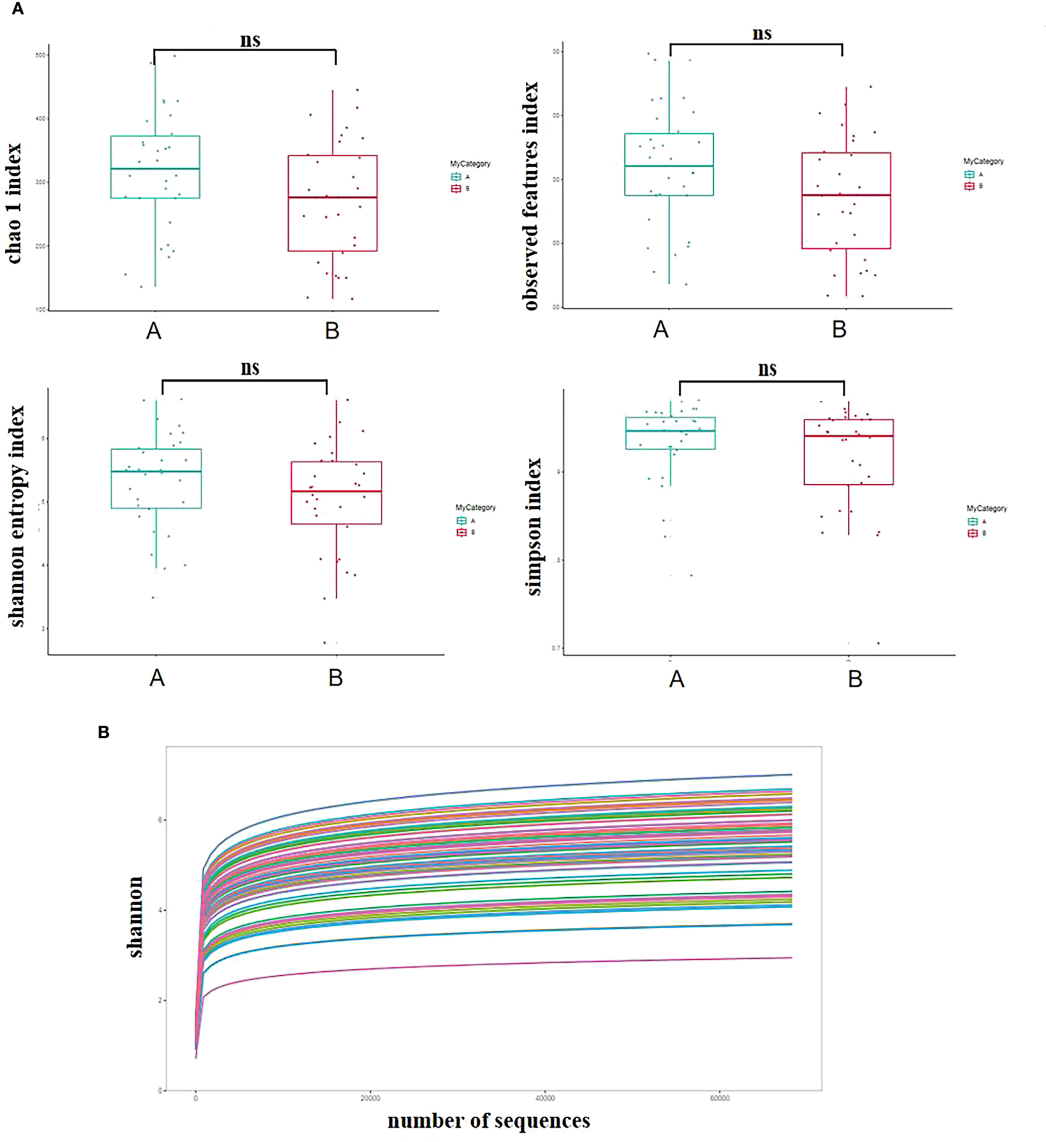
Comparison of alpha diversity in gut microbiota. **(A)** compares the alpha diversity (Chao1, Observed species, Shannon, Simpson) indices between the two groups; **(B)** the sampling depth on the x-axis and the Shannon index on the y-axis. “ns” indicates no statistical significance, Group A: healthy group, Group B: AH group.

As shown in **Figure 2A**, the Chao1 index, Observed species index, Shannon index, and Simpson index of the AH group were lower than those of the healthy control group, but the differences were not statistically significant (P > 0.05). As depicted in **Figure 2B** below, the sequencing depth of the experimental samples gradually reached saturation with increasing sequencing efforts, indicating sufficient sequencing coverage to assess the diversity of the gut microbiota under study.

### 3.4 Comparison of beta diversity in gut microbiota

**Figure 3:**
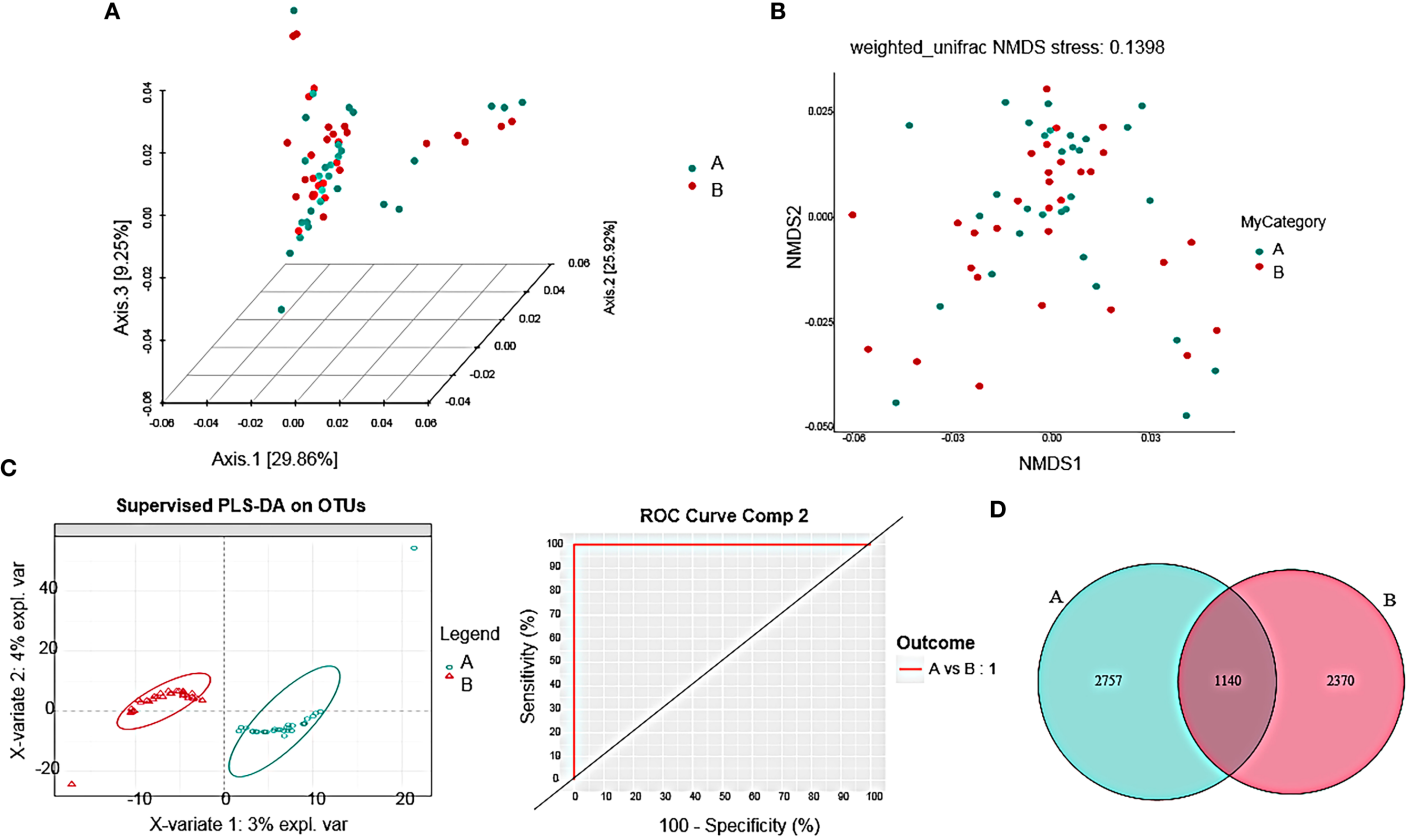
Comparison of beta diversity in gut microbiota. **(A)** Based on the 3D PCoA plot using weighted Unifrac, Axis 1 represents the first principal component contributing 29.86%, while Axis 2 represents the second principal component contributing 9.25%. **(B)** NMDS analysis was conducted using weighted Unifrac distances. Each point in the plot represents a sample, with different colors indicating different sample groups, and the distances between points represent the degree of microbial community differences. **(C)** PLS-DA coordinate plot where each point represents a sample, with points of the same color belonging to the same group; AUC curves of the regression model indicating the area under the curve for discriminant analysis of each group displayed on the right side of the plot. **(D)** Venn diagram showing Group A: healthy group, Group B: AH group.

The PCoA analysis (**Figure 3A**) revealed differences in the gut microbiota community structure between the AH group and the healthy control group. Further confirmation of these differences was obtained through ANOSIM analysis, which indicated non-significant dissimilarities between the two groups (P=0.69). NMDS analysis results were consistent with the PCoA analysis (**Figure 3B**). Utilizing Partial Least Squares Discriminant Analysis (PLS-DA) (**Figure 3C**), the samples from the two groups were distinctly separated in the PLS-DA plot without overlap, with an AUC value of 1 for both groups, indicating significant differences in gut microbiota composition between them. The Venn diagram (**Figure 3D**) visually displayed the shared and unique species compositions between the healthy and AH groups, showing 1140 shared ASVs and a differing number of ASVs, highlighting a quantitative difference in species between the two groups.

### 3.5 LEfSe analysis of divergent species

**Figure 4:**
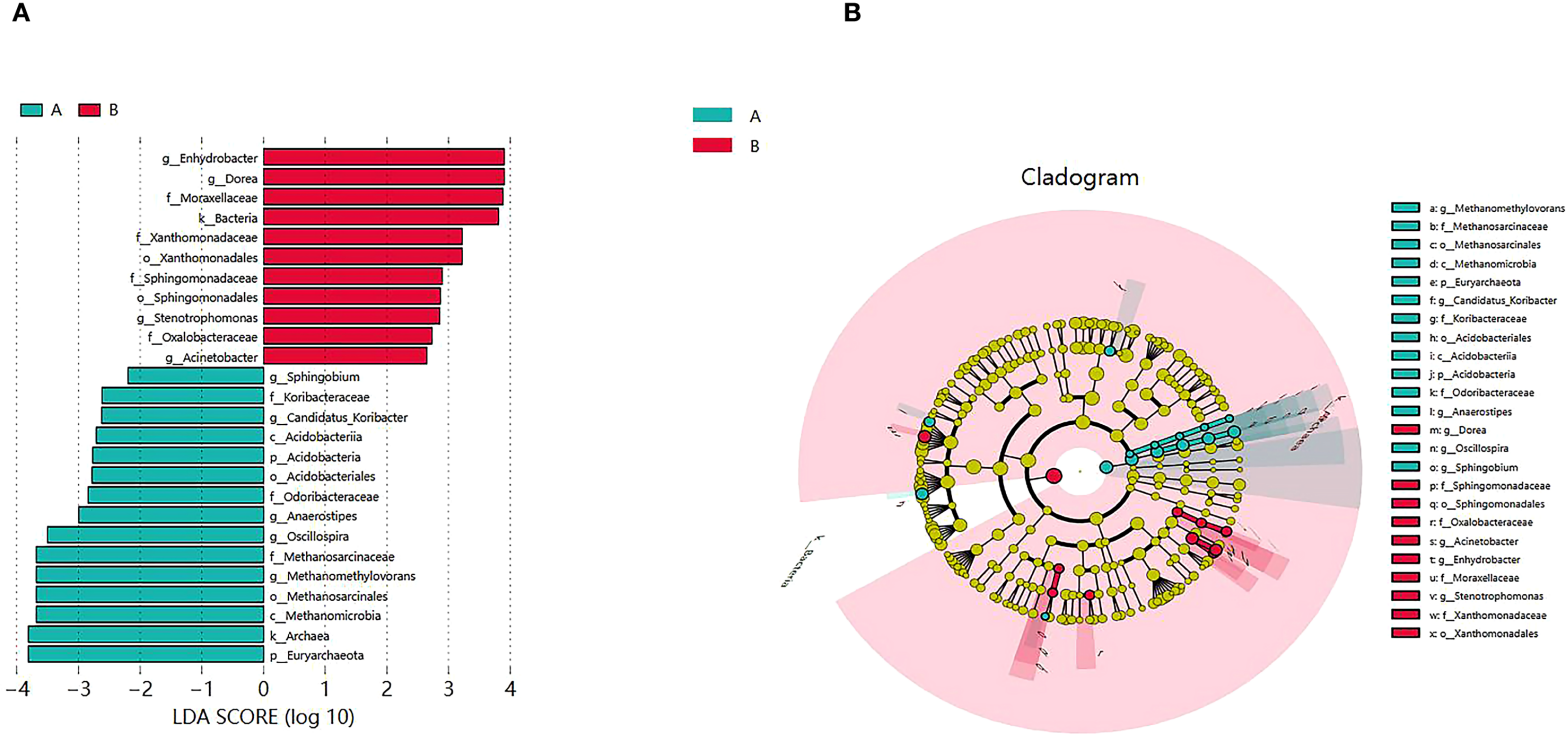
Analysis of differential species in the gut microbiota. **(A)** Each horizontal bar represents a species, with the length corresponding to the LDA value indicating the level of difference. The color of the bars indicates the microbial feature group to which the species belongs, reflecting its relatively higher abundance within that group. **(B)** In the cladogram, the layers from inner to outer represent different taxonomic levels such as phylum, class, order, family, and genus, with connecting lines indicating their hierarchical relationships. Each circular node represents a species, with yellow nodes indicating insignificant differences between groups, while non-yellow nodes represent species characteristic of a specific group (with significantly higher abundance within that group). Colored sectors highlight the taxonomic range of the characteristic microbes. Group A: healthy group, Group B: AH group.

As shown in **Figure 4**, compared to the healthy group, the AH group exhibited higher relative abundance of g_Enhydrobacter, g_Dorea, g_Stenotrophomonas, and g_Acinetobacter (P < 0.05), while g_Sphingobium, g_Candidatus_Koribacter, p_Acidobacteria, g_Anaerostipes, g_Oscillospira, g_Methanomethylovorans, and p_Euryarchaeota showed lower relative abundance (P < 0.05).

### 3.6 Correlation analysis

**Figure 5:**
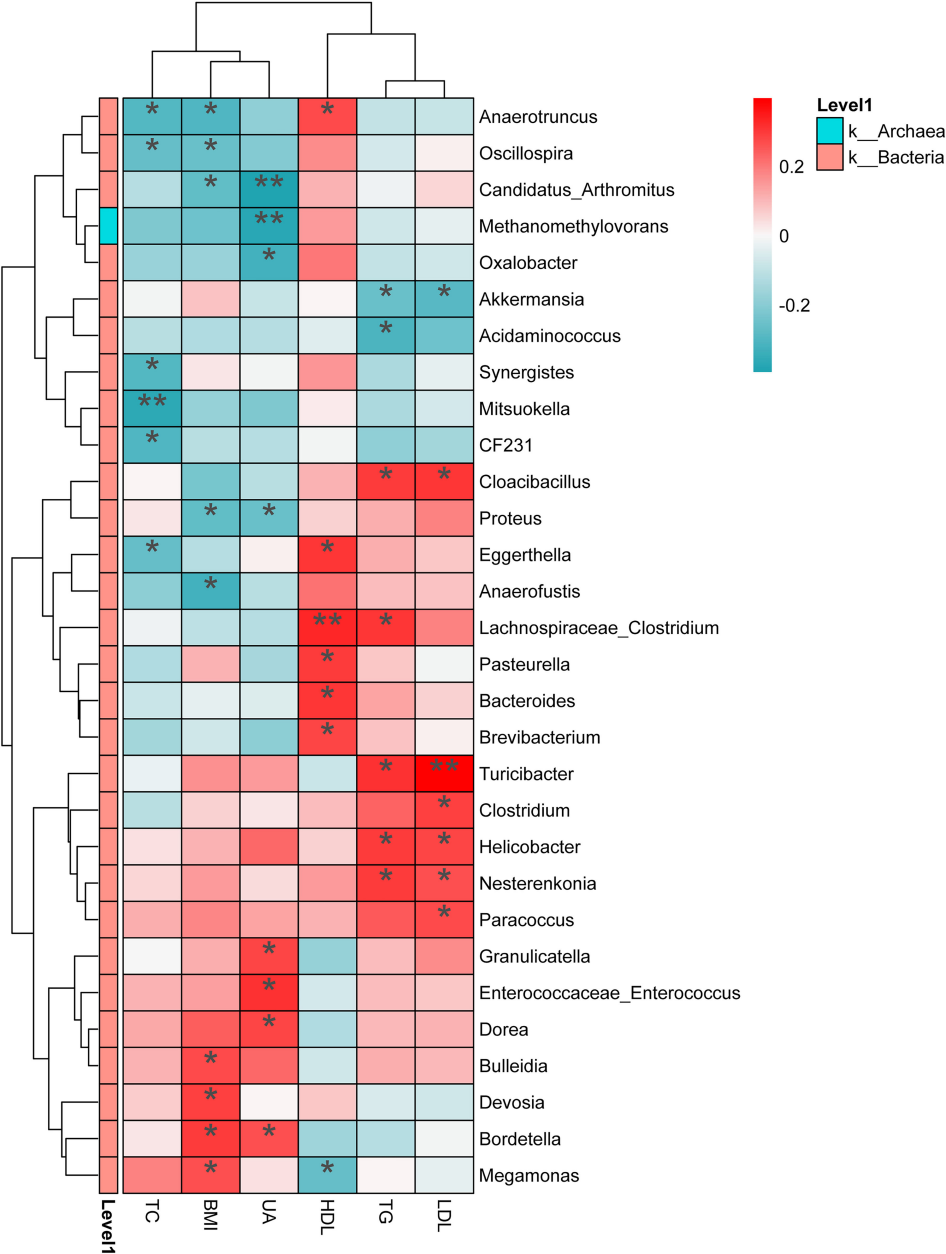
Heatmap illustrating the relationships between microbial species and clinical phenotypes in the study participants (r ≥ 0.3). The X-axis represents clinical phenotypes (including BMI, TC, TG, LDL-C, UA, HDL-C), while the Y-axis indicates the corresponding species (at the genus level). The color bar on the far left denotes the phylum classification, with different colors representing different r values. The legend on the far right shows the color intervals corresponding to r values and the phylum names. *P < 0.05, **P < 0.01. BMI, Body Mass Index; TC, Total Cholesterol; TG, Triglycerides; LDL-C, Low-Density Lipoprotein Cholesterol; HDL-C, High-Density Lipoprotein Cholesterol; UA, Serum Uric Acid.

The Spearman correlation analysis revealed a positive correlation between blood uric acid levels and the abundance of Enterococcaceae, Dorea, Bordetella, and Granulicatella (P < 0.05), and a negative correlation with Oxalobacter, Methanomethylovorans, Candidatus_Arthromitus, and Proteus (P < 0.05) (**Figure 5**). Additionally, clinical parameters such as BMI and blood lipid levels showed significant correlations with the abundance of certain gut microbial species.

### 3.7 Analysis of inter-group differences in functionality

**Figure 6:**
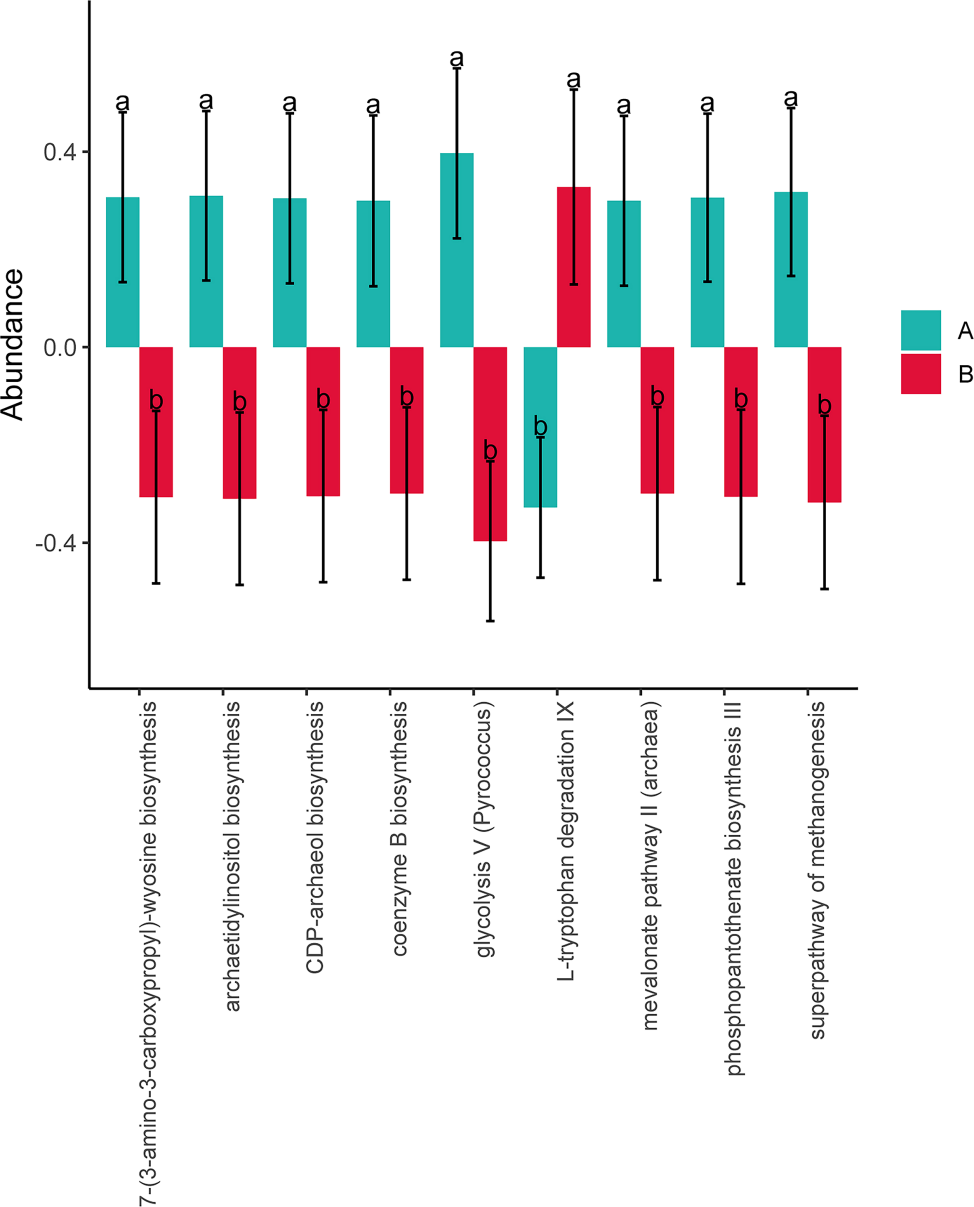
Significant differences in all METAcyc pathways identified through ANOVA and Duncan’s test. The x-axis represents the names of pathways. Each pathway is color-coded to indicate a specific group. If two groups share the same letter above them, it signifies nonsignificant differences; otherwise, differences are considered significant.

Based on PICRUSt2-predicted pathway annotations (MetaCyc database) and considering the grouping information, microbial community predicted functions were analyzed using ANOVA with Duncan and Dunn tests. As shown in **Figure 6**, the AH group exhibited downregulation of glycolysis V (Pyrococcus), 7-(3-amino-3-carboxypropyl)-wyosine biosynthesis, archaetidylinositol biosynthesis, CDP-archaeol biosynthesis, coenzyme B biosynthesis, mevalonate pathway II (archaea), phosphopantothenate biosynthesis III, and superpathway of methanogenesis, while L-tryptophan degradation IX was upregulated compared to the healthy group, with significant differences (P < 0.05).

## 4 Discussion

This study explores the background of AH and its potential associations with metabolic-related diseases. AH is characterized by elevated blood uric acid levels without symptoms of conditions like gout. Despite a rising prevalence in the general population and its close links to health risks such as cardiovascular diseases, kidney stones, and metabolic syndrome, research on AH remains limited, particularly regarding its underlying pathophysiology and preventive strategies. This suggests that AH may serve as a precursor to more serious health issues, emphasizing the need for further investigation in this field.

This study investigates the observed association between changes in gut microbiota and AH. Various methods including clinical data collection, high-throughput sequencing, and microbiome data analysis were employed to analyze the gut microbiota characteristics of AH patients compared to healthy controls, and their relationship with clinical indicators. The results reveal significant differences in gut microbiota composition between AH patients and healthy individuals, suggesting new targets for future treatments. Through a thorough analysis of these findings, we aim to provide novel insights and evidence for the prevention and treatment of AH.

In this study, we revealed a potential association between AH and the gut microbiota by analyzing the characteristics of AH patients and their gut microbiomes. The results indicated significant variances between the AH group and the healthy control group in terms of body mass index (BMI) and metabolic indicators such as TG and TC. These variances suggest distinct metabolic features in AH patients compared to healthy individuals, providing foundational data for clinical screening and intervention strategies. Additionally, the prevalence of hypertension and fatty liver in the AH group was significantly higher than in the healthy group, further supporting a potential link between AH and metabolic syndrome (8).

In the analysis of microbiota data, this study observed a decrease in Alpha diversity in the AH group compared to the healthy group, indicating a reduction in overall richness and ecological diversity of the gut microbiota in the AH group. Beta diversity suggested differences in the composition of gut microbiota between the two groups. At the phylum level, a decrease in abundance of Acidobacteria and Verrucomicrobia was noted in the AH group. At the genus level, the relative abundance of Enhydrobacter, Dorea, Stenotrophomonas, Acinetobacter was higher, while Sphingobium, Candidatus_Koribacter, Anaerostipes, Oscillospira, Methanomethylovorans showed lower relative abundance in the AH group. These microbial differences may be correlated with the pathogenesis of AH, providing clues for future research on the relationship between gut microbiota and AH. Previous studies have suggested that gut microbiota are associated with uric acid synthesis and excretion through metabolic pathways and immune responses (9).

Some studies suggest that Oscillospira is a genus associated with a healthy gut and has been predicted to be a potential producer of butyric acid (10). Anaerostipes strains are known to utilize inositol to generate propionic and acetic acids (11). SCFAs, particularly propionate and butyrate, have been reported to provide ATP to intestinal cells, potentially benefiting uric acid excretion (12). Our research also observed decreased abundance of Oscillospira and Anaerostipes in the AH group, indicating a possible association between AH and the reduction of these two genera.

Furthermore, the study revealed a positive correlation between serum uric acid levels in AH patients and specific gut microbiota such as Bordetella and Granulicatella, while showing a negative correlation with Oxalobacter and Methanomethylovorans (P < 0.05). These findings offer a novel perspective for biomarker research in AH, potentially aiding in early diagnosis and monitoring in clinical settings. Future research should explore the utility of microbiome analysis techniques for early diagnosis and investigate the prospects of these biomarkers in personalized medicine (13).

Finally, based on the PICRUSt2-predicted pathway abundances annotated by the METAcyc database, we observed significant differences in specific metabolic pathways, including downregulation of glycolysis V (Pyrococcus) and upregulation of L-tryptophan degradation IX in the AH group compared to the healthy group. However, as these functional predictions are derived from 16S rRNA gene sequences and not from metagenomic or metatranscriptomic data, they should be interpreted as in silico inferences with inherent limitations including dependence on reference genomes and lack of experimental validation.

Despite these predictive limitations, previous research has provided insights that may relate our findings to hyperuricemia pathogenesis. Potential inhibition of the glycolysis pathway could theoretically contribute to accumulation of metabolic intermediates such as 6-phospho-glucose and 3-phosphoglyceraldehyde, which can be diverted to produce 5-phosphoribose and subsequently ribose-5-phosphate. This could theoretically increased endogenous purine synthesis and elevated serum uric acid levels (14). It is noteworthy that the predicted glycolysis V pathway is specific to archaea (Pyrococcus), which implies a possible connection of archaeal metabolism in AH that warrants further investigation.

Regarding tryptophan metabolism, which encompasses the kynurenine, serotonin, and indole pathways, the production of bioactive compounds from tryptophan degradation can be associated with diverse physiological processes including inflammation, metabolism, and immune responses (15). Kynurenic acid, a major degradation product of L-tryptophan (16), was found elevated in AH rats, while indoxyl sulfate and tryptophan 2-C-glucoside were decreased, indicating alterations in tryptophan metabolism in AH (17). Although our predictive data suggest altered tryptophan degradation activity in the gut microbiome of AH patients, the nature of the relationship between microbial pathway activity and host metabolite levels requires experimental validation.

The limitations of this study primarily lie in sample size and experimental design. Although we compared high uric acid patients with a healthy control group, the relatively small sample size may impact the statistical significance and generalizability of the results. Furthermore, the observational design prevents causal inferences, and the lack of validation through wet lab experiments restricts the biological significance and clinical applicability of the findings. Additionally, important lifestyle factors including dietary patterns, fiber intake, physical activity, and probiotic use were not assessed in this study. These unmeasured confounders may influence both gut microbiota composition and uric acid levels, limiting the ability to isolate microbiome-specific effects. Moreover, the absence of long-term clinical follow-up data prevents the assessment of potential causality between microbiome changes and the progression of AH. These limitations suggest that future research should involve larger sample sizes, incorporate multiple experimental approaches, control for key lifestyle confounders, and include long-term follow-up to validate our findings and enhance their clinical relevance.

In conclusion, this study provides important foundational data for clinical management by analyzing the characteristics of patients with AH, differences in gut microbiota, and associated risk factors. Despite certain limitations, our findings suggest new research directions for early diagnosis and personalized treatment of AH. Future investigations into the relationship between microbiota and AH, combined with larger-scale clinical data, may contribute to the development of more effective prevention and treatment strategies.

## Data Availability

The datasets presented in this study can be found in online repositories.The data presented in this study are deposited in the NCBI Sequence Read Archive (SRA) repository https://www.ncbi.nlm.nih.gov/sra/, accession number PRJNA1336442.
